# Thanks to all those who reviewed for *Systematic Reviews* in 2014

**DOI:** 10.1186/s13643-015-0003-9

**Published:** 2015-02-21

**Authors:** David Moher, Paul Shekelle, Lesley A Stewart

**Affiliations:** Ottawa Hospital Research Institute and University of Ottawa, Ottawa, Canada; Southern California Evidence-based Practice Center, Santa Monica, CA USA; Centre for Reviews and Dissemination, University of York, York, UK

## Abstract

A peer-reviewed journal would not survive without the generous time and insightful comments of the reviewers, whose efforts often go unrecognized. Although final decisions are always editorial, they are greatly facilitated by the deeper technical knowledge, scientific insights, understanding of social consequences, and passion that reviewers bring to our deliberations. For these reasons, the Editors-in-Chief and staff of the journal warmly thank the 219 reviewers whose comments helped to shape *Systematic Reviews*, for their invaluable assistance with review of manuscripts for the journal in Volume 3 (2014).

**Jawdat Abdulla**

Denmark

**Clive E. Adams**

UK

**Myzoon Ali**

UK

**Eitan Amir**

Canada

**Raghupathy Anchala**

India

**Lisa Askie**

Australia

**Marc Avey**

Canada

**Ethan Balk**

USA

**Joel Bamford**

USA

**David Bellinger**

USA

**Jennifer Bellis**

UK

**Kate Bennett**

UK

**Nancy Berkman**

USA

**Jesse Berlin**

USA

**Laurent Beydon**

France

**Unnikrishnan Bhaskaran**

India

**Zhaoxiang Bian**

Hong Kong

**John Billi**

USA

**Giuseppe Biondi-Zoccai**

Italy

**Jan Blaakaer**

Denmark

**Stefania Boccia**

Italy

**Laura Bonnett**

UK

**Jonathan Boote**

UK

**Andrew Booth**

UK

**Isabelle Boutron**

France

**Caroline Braet**

Belgium

**Thomas Butt**

UK

**Deborah Caldwell**

UK

**Alun Cameron**

Australia

**Chris Cameron**

Canada

**Fiona Campbell**

UK

**Kristin Carson**

Australia

**Adriana Castelli**

UK

**Ferrán Catalá-López**

Spain

**Yaping Chang**

Canada

**Deepak Chawla**

India

**Hui-Ching Chuang**

Taiwan

**Americo Cicchetti**

Italy

**Shannon Cope**

Canada

**Alessandro Coppo**

Italy

**Miranda Cumpston**

Australia

**Huw Davies**

UK

**Cinzia del Giovane**

Italy

**Vakaramoko Diaby**

USA

**Sofia Dias**

UK

**Kelly Dickson**

UK

**Therese Dowswell**

UK

**Janine Dretzke**

UK

**Isabelle Durand-Zaleski**

France

**Kerry Dwan**

UK

**Donald Edmondson**

USA

**Regina El Dib**

Brazil

**Alexandra Ellis**

USA

**Louise Falzon**

USA

**Forough Farrokhyar**

Canada

**Katalin Fekete**

Hungary

**Jonas David Finger**

Germany

**Alan Fleischer**

USA

**Kate Flemming**

UK

**Rosanne Freak-Poli**

Australia

**Tomohiro Funakoshi**

USA

**James Galipeau**

Canada

**Rebecca Ganann**

Canada

**Ruth Garside**

UK

**Gerald Gartlehner**

Austria

**Charlotte Gaydos**

USA

**Klaus Gebel**

Australia

**Su Golder**

UK

**Sean Grant**

UK

**Katja Grasic**

UK

**Janette Greenhalgh**

UK

**Trisha Greenhalgh**

UK

**Kelly Grindrod**

Canada

**Ulrich Grouven**

Germany

**Annetje Guedon**

Netherlands

**Ruth Gwernan-Jones**

UK

**Susanne Hempel**

USA

**William Hersh**

USA

**Renee Hessian**

Canada

**Suzanne Hill**

Australia

**Caroline Homer**

Australia

**Min Kyung Hyun**

South Korea

**Justin Jagosh**

Canada

**Nikki Jahnke**

UK

**Heethal Jaiprakash**

Malaysia

**Anthony James**

UK

**Wayne Jonas**

USA

**Cassandra Josephson**

USA

**Robert Kane**

USA

**Marloes Kleinjan**

Netherlands

**Levente Kriston**

Germany

**Oliver Kuss**

Germany

**William Lambert**

USA

**Toby Lasserson**

UK

**Mariska M. G. Leeflang**

Netherlands

**Anne Lethaby**

New Zealand

**James Lewis**

USA

**Yuan Li**

China

**Tianjing Li**

USA

**Kuancho Liao**

Taiwan

**Helen Liley**

Australia

**Yu-Tsai Lin**

Taiwan

**Kathleen Lohr**

USA

**Yoon Kong Loke**

UK

**Frank Louws**

USA

**Georgios Lyratzopoulos**

UK

**Laxmaiah Manchikanti**

USA

**Ana Marusic**

Croatia

**Annette Matthews**

USA

**Alain Mayhew**

Canada

**Evan Mayo-Wilson**

USA

**Lawrence Mbuagbaw**

Cameroon

**Catriona McDaid**

UK

**Marian McDonagh**

USA

**G. J. Melendez-Torres**

UK

**Virginia Minogue**

UK

**Suneeta Mittal**

India

**Kaelan Moat**

Canada

**David Moher**

Canada

**Ali Montazeri**

Iran

**Horieh Moosavi**

Iran

**Manuel Muñoz**

Spain

**Michael Murphy**

UK

**Huseyin Naci**

UK

**Sreekumaran Nair**

India

**Suma Nair**

India

**Sydne Newberry**

USA

**Mark Newman**

UK

**Lisbeth Nilas**

Denmark

**Sarah Nolan**

UK

**Susan L. Norris**

USA

**Allison Ober**

USA

**Stephen O'Leary**

Australia

**Wendy Olsen**

UK

**Maya O'Neil**

USA

**Michael Ostacher**

USA

**Carrie Patnode**

USA

**Porjai Pattanittum**

Thailand

**Mark Pearson**

UK

**Einat Peles**

Israel

**Bob Phillips**

UK

**Julie Polisena**

Canada

**Jennie Popay**

UK

**Stephanie Prince**

Canada

**Marsha Raebel**

USA

**Rajeev Ramchand**

USA

**Andrew Rankin**

UK

**H Fisher Raymond**

USA

**Barnaby Reeves**

UK

**Toby Richards**

UK

**David Riley**

USA

**Emilie Robert**

Canada

**Mark Rodgers**

UK

**Martin Rohling**

USA

**Maroeska Rovers**

Netherlands

**Gerta Rücker**

Germany

**George Rutherford**

USA

**Laura Sampietro-Colom**

Spain

**Margaret Sampson**

Canada

**Connie Schardt**

USA

**Roberta Scherer**

USA

**David Schriger**

USA

**Jodi Segal**

USA

**Shelley Selph**

USA

**Tatyana Shamliyan**

USA

**Larissa Shamseer**

Canada

**Beverley Shea**

Canada

**Paul Shekelle**

USA

**Nathan Shippee**

USA

**Vijay Shukla**

Canada

**Jean Sibilia**

France

**Becky Skidmore**

Canada

**Rebecca Smyth**

UK

**Andy Spencer**

UK

**Ann Sprague**

Canada

**Robert Stanton**

Australia

**Adrienne Stevens**

Canada

**Kitty Stewart**

UK

**Lesley Stewart**

UK

**Catalina Suarez-Cuervo**

USA

**Carolyn Summerbell**

UK

**Yemisi Takwoingi**

UK

**Howard Thom**

UK

**Carl Thompson**

UK

**Hilary Thomson**

UK

**Judith Thornton**

UK

**Jin-Hui Tian**

China

**Guy Tsafnat**

Australia

**Tony Tse**

USA

**Alexander Tsertsvadze**

Canada

**Ioannis Tzanakis**

Greece

**Jelle van Cauwenberg**

Belgium

**Wynanda van Enst**

Netherlands

**Gert van Valkenhoef**

Netherlands

**Andre Veras**

Brazil

**Areti Angeliki Veroniki**

Canada

**Liat Vidal**

Israel

**Jean-Louis Vincent**

Belgium

**John Wahren**

Sweden

**Byron Wallace**

USA

**Rebecca Waller**

USA

**Debbie Warman**

USA

**Vivian Welch**

Canada

**Nicky Welton**

UK

**Suzanne West**

USA

**Gill Westhorp**

Australia

**Marie Elaine Westwood**

UK

**Penny Whiting**

UK

**Evelyn Whitlock**

USA

**Craig Whittington**

UK

**Kumanan Wilson**

Canada

**Paul M. Wilson**

UK

**Michael Wilson**

Canada

**Nicole Wolfe**

USA

**Geoff Wong**

UK

**Judy Wright**

UK

**Kath Wright**

UK

**Po-Yin Yen**

USA

**Junhua Zhang**

China

