# Erratum to: Thanks to all those who reviewed for systematic reviews in 2014

**DOI:** 10.1186/s13643-015-0087-2

**Published:** 2015-07-30

**Authors:** David Moher, Paul Shekelle, Lesley A. Stewart

**Affiliations:** Ottawa Hospital Research Institute and University of Ottawa, Ottawa, Canada; Veterans Affairs Greater Los Angeles Health Care System, Los Angeles, ᅟ ᅟ; RAND Health, Santa Monica, ᅟ ᅟ; Centre for Reviews and Disseminationcs, University of York, York, UK

## Erratum

Unfortunately, the original version of this article [1] published with an incorrect citation. The citation has now been updated to 4:94, which is the correct citation for the article. The Publisher apologizes for any inconvenience caused.

## References

[1] Moher D, Shekelle P, Stewart LA (2015). Thanks to all those who reviewed for Systematic Reviews in 2014. Syst Rev.

